# A semi-supervised boosting SVM for predicting hot spots at protein-protein Interfaces

**DOI:** 10.1186/1752-0509-6-S2-S6

**Published:** 2012-12-12

**Authors:** Bin Xu, Xiaoming Wei, Lei Deng, Jihong Guan, Shuigeng Zhou

**Affiliations:** 1 Department of Computer Science and Technology, Tongji University, Shanghai 201804, China; 2 Shanghai Key Lab of Intelligent Information Processing, and School of Computer Science, Fudan University, Shanghai 200433, China

## Abstract

**Background:**

Hot spots are residues contributing the most of binding free energy yet accounting for a small portion of a protein interface. Experimental approaches to identify hot spots such as alanine scanning mutagenesis are expensive and time-consuming, while computational methods are emerging as effective alternatives to experimental approaches.

**Results:**

In this study, we propose a semi-supervised boosting SVM, which is called sbSVM, to computationally predict hot spots at protein-protein interfaces by combining protein sequence and structure features. Here, feature selection is performed using random forests to avoid over-fitting. Due to the deficiency of positive samples, our approach samples useful unlabeled data iteratively to boost the performance of hot spots prediction. The performance evaluation of our method is carried out on a dataset generated from the ASEdb database for cross-validation and a dataset from the BID database for independent test. Furthermore, a balanced dataset with similar amounts of hot spots and non-hot spots (65 and 66 respectively) derived from the first training dataset is used to further validate our method. All results show that our method yields good sensitivity, accuracy and F1 score comparing with the existing methods.

**Conclusion:**

Our method boosts prediction performance of hot spots by using unlabeled data to overcome the deficiency of available training data. Experimental results show that our approach is more effective than the traditional supervised algorithms and major existing hot spot prediction methods.

## Background

Protein-protein interactions (PPIs) are critical for almost all biological processes [1-3]. Many efforts have been made to investigate the residues at protein-protein interfaces. The checking of a large number of protein-protein interaction interfaces has shown that there are no general rules, which can describe the interfaces precisely [4-10]. It is also well known that the binding free energy is not uniformly distributed over the protein interfaces, and a small portion of interface residues contribute the most of binding free energy instead [11]. These residues are termed as *hot spots*. Identifying hot spots and revealing their mechanisms may provide promising prospect for medicinal chemistry.

Alanine-scanning mutagenesis [12] is a popular method to identify hot spots by evaluating the change in binding free energy when substituting interface residues with alanine. Hot spots are defined as those sites where alanine mutations cause a significant change in binding free energy (ΔΔ*G*). Owing to the high cost and low efficiency of this traditional experimental method, public databases of experimental results such as the Alanine Scanning Energetics Database (ASEdb) [13] and the Binding Interface Database (BID) [14] contain only a limited number of complexes.

Some works focused on the characteristics of hot spot due to its critical role. Studies on the composition of hot spots and non-hot spots have revealed that Trp, Arg and Tyr rank the top 3, with the rates of 21%, 13.3% and 12.3% respectively. While Leu, Ser, Thr and Val are often disfavored [15,16]. Furthermore, hot spots are found to be more conserved than non-hot spots, and they are usually surrounded by a group of residues not important for binding, whose role is to shelter hot spots from the solvent [17].

Based on the existing studies on the characteristics of hot spots, some computational methods have been proposed to predict hot spots. These methods roughly fall into three categories: molecular dynamics (MD) simulations, energy-based methods and feature-based methods.

Molecular dynamics (MD) [18-20] simulations simulate alanine substitutions and estimate the corresponding changes in binding free energy. Although these molecular simulation methods have good performance on identifying hot spots from protein interfaces, they suffer from enormous computational cost.

Energy-based methods use knowledge-based simplified models to evaluate binding free energy for predicting hot spots. Kortemme and Baker [21] proposed a simple physical model using a free energy function to calculate the binding free energy of alanine mutation in a protein-protein complex. Guerois et al., [22] provided FOLDEF whose predictive power has been tested on a large set of 1088 mutants spanning most of the structural environments found in proteins. Tuncbag et al., [23] established a web server Hotpoint combining conservation, solvent accessibility and statistical pairwise residue potentials to computationally predict hot spots effectively.

In recent years, some machine learning based methods with focus on feature selection were developed to identify hot-spots. Ofran and Rost [24] proposed a neural network based on sequence to predict hot spots. Darnell et al., [25] provided a web server KFC by using decision trees to predict hot spots. Some works use different features as input of a Support Vector Machine (SVM) classifier to predict hot spots. Cho et al., [26] developed two feature-based predictive SVM models for predicting interaction hot spots. Xia et al., [27] introduced both a SVM model and an ensemble classifier based on protrusion index and solvent accessibility to boost hot spots prediction accuracy. Zhu and Mitchell [28] developed a new web server, named KFC2, by employing SVM with some newly derived features.

Although machine learning based methods have obtained relatively good performance on the prediction of hot spots. There are still some problems remaining in this area. Though many features have been generated and used in the previous studies, effective feature selection methods and useful feature subsets have not been found yet. Moreover, most of the existing methods use very limited data from experiment-derived deposits, therefore the training set is insufficient, which leads to unsatisfactory prediction performance.

To deal with the problems mentioned above, in this paper we first extract features of both sequence and structure, and employ random forests [29] to generate an effective feature subset. Then we propose a boosting SVM based approach, sbSVM, to improve the prediction of hot spots by using unlabeled data. Our method integrates unlabeled data into the training set to overcome the problem of labeled data inadequacy. Finally, we evaluate the proposed method by 10-fold cross-validation and independent test, which demonstrate the performance advantage of our approach over the existing methods.

## Methods

### Datasets

The first training data set in this study, denoted as *dataset*1, was extracted from ASEdb [13] and the published data by Kortemme and Baker [21]. To eliminate redundancy, we used the CATH (Class (C), Architecture (A), Topology (T) and Homologous superfamily (H)) query system with the sequence identity less than 35% and the SSAP score less than or equal to 80. Details are listed in Table 1. We define interface residues with ΔΔ*G ≥ *2.0 kcal/mol as hot spots and those with ΔΔ*G ≤ *2.0 kcal/mol as non-hot spots [26,28,30].

**Table 1:** The details of *dataset*1.

PDB	1st Molecule	2nd Molecule	H	NH
1a4y	Angiogenin	Ribonuclease Inhibitor	3	12

1a22	Human growth hormone	Human growth hormone binding protein	7	29

1ahw	Immunoglobulin Fab 5G9	Tissue factor	1	3

1brs	Barnase	Barstar	8	1

1bxi	Colicin E9 Immunity Im9	Colicin E9 DNase	6	3

1cbw	BPTI Trypsin inhibitor	Chymotrypsin	1	4

1dan	Blood coagulation factor VIIA	Tissue factor	2	9

1dvf	Idiotopic antibody FV D1.3	Anti-idiotopic antibody FV E5.2	6	1

1fc2	Fc fragment	Fragment B of protein A	1	0

1fcc	Fc (IGG1)	Protein G	4	2

1gc1	Envelope protein GP120	CD4	0	11

1jrh	Antibody A6	Interferon-gamma receptor	8	5

1vfb	Mouse monoclonal antibody D1.3	Hen egg lysozyme	3	6

2ptc	BPTI	Trypsin	1	0

3hfm	Hen Egg Lysozyme	lg FAB fragment HyHEL-10	11	6

**H stands for Hot Spot and NH stands for Non-Hot Spot**. *Dataset*2 **was derived from ***dataset*1.

As a result, *dataset*1 consists of 265 interface residues derived from 17 protein-protein complexes, where 65 residues are hot spots and 200 residues are energetically unimportant residues. In order to train better predictors, we balanced the positive and negative samples as in [28]. The negative samples (non-hot spots) were divided into 3 groups and each was combined with the positive samples (hot spots). The third group (66 non-hot spots) combines with 65 hot spots, which is denoted as *dataset*2 and can obtain better results than the other two combinations when being used to train our predictor.

An independent test dataset, denoted as *ind*-*dataset*, was obtained from the BID database [14] to further evaluate our method. In the BID database, the alanine mutations were listed as either "strong", "intermediate", "weak" or "insignificant". In this study, only residues with "strong" mutations are considered as hot spot and the others are regarded as non hot spot. As a result, *ind*-*dataset *consists of 126 interface residues derived from 18 protein-protein complexes, where 39 residues are hot spots and 87 residues are energetically unimportant residues.

As a summary, the statistics of *dataset*1, *dataset*2 and *ind*-*dataset *are presented in Table 2.

**Table 2:** Statistics of *dataset*1, *dataset*2 and *ind*-*dataset*.

Dataset	Number of hot spots	Number of non-hot spots	Total number
*dataset*1	65	200	265

*dataset*2	65	66	131

*ind-dataset*	39	87	126

### Features

Based on previous studies on hot spots prediction, we generate 6 sequence features and 62 structure features.

#### Sequence features

The sequence features used in this paper include the number of atoms, electron-ion interaction potential, hydrophobicity, hydrophilicity, propensity and isoelectric point. These physicochemical features can be obtained from the AAindex database [31].

#### Structure features

Firstly, we used the implementation PSAIA proposed by Mihel et al., [32] to generate features about solvent accessible surface area (ASA), relative solvent accessible surface area (RASA), depth index (DI) and protrusion index (PI), which are defined as follows:

• Accessible surface area (ASA, usually expressed in *Å*_2_) is the atomic surface area of a molecule, protein and DNA etc., which is accessible to a solvent.

• Relative ASA (RASA) is the ratio of the calculated ASA over the referenced ASA. The reference ASA of a residue X is obtained by Gly-X-Gly peptide in extended conformations [33].

• Depth index (DI): the depth of an atom *i *(*DPXi*) can be defined as the distance between atom *i *and the closest solvent accessible atom *j*. That is, *DPXi *= min(*d*_1_, *d*_2_, *d*_3_, ..., *d_n_*) where *d*_1_, *d*_2_, *d*_3_, ..., *d_n _*are the distances between the atom *i *and all solvent accessible atoms.

• Protrusion index (PI) is defined as *V_ext_*/*V_int_*. Here, *V_int _*is given by the number of atoms within the sphere (with a fixed radius *R*) multiplied by the mean atomic volume found in proteins; *V_ext _*is the difference between the volume of the sphere and *V_int_*, which denotes the remaining volume of the sphere.

From ASA and RASA, five attributes can be derived:

• total (the sum of all atom values);

• backbone (the sum of all backbone atom values);

• side-chain (the sum of all side-chain atom values);

• polar (the sum of all oxygen, nitrogen atom values);

• non-polar (the sum of all carbon atom values).

And based on DI and PI, four residue attributes can be obtained:

• total mean (the mean value of all atom values);

• side-chain mean (the mean value of all side-chain atom values);

• maximum (the maximum of all atom values);

• minimum (the minimum of all atom values).

Therefore, 36 features were generated by PSAIA from unbound and bound states.

In addition, the relative changes of ASA, DI and PI between the unbound and bound states of the residues were calculated as in Xia et al's work [27], and 13 more features were generated by the equations below:

$$\begin{array}{c} RcASA=\left(ASA_{unbound}-ASA_{bound}\right)/ASA_{unbound},\\ RcDI=\left(DI_{bound}-DI_{unbound}\right)/DI_{bound},\\ RcPI=\left(PI_{unbound}-PI_{bound}\right)/PI_{unbound}. \end{array}$$

Furthermore, we generated some useful features following the strategy of KFC2 [28]. Residues' solvent accessible surface is used in the following features and is calculated by NACCESS [34].

*DELTA_TOT *describes the difference between the solvent accessible surfaces in bound and unbound states:

DELTA_TOT=ASAunb-ASAbnd.

*SA_RATIO*5 is the ratio of solvent accessible surface area over maxASA, which stands for the residue's maximum solvent accessible surface area as a tripeptide [35]:

$$SA\text{\_}RATIO5=\frac{DELTA\text{\_}TOT\times maxASA}{ASAunb}.$$

Another form of ratio of solvent accessible surface area, *CORE_RIM*, is given by:

$$CORE\text{\_}RIM=\frac{DELTA\text{\_}TOT}{ASAunb}.$$

and this feature is quite like *the relative change in total ASA *described before. The main difference lies in that PSAIA treats each chain separately during the calculation [32]. In our work we will use at most one of these two features in order to avoid a bias.

*POS_PER *is defined as below, where *i *is the sequence number of the residue and *N *is the total number of the interface residues:

$$POS\text{\_}PER=CORE\text{\_}RIM\times \frac{i}{N}.$$

*ROT*4 and *ROT*5 stand for the total numbers of the side chain rotatable single bonds to target residues for the residues within 4.0Å and 5.0 Å, respectively.

HP5 is the sum of hydrophobic values of all neighbors of a residue within 5Å.

FP9N, FP9E, FP10N and FP10E were directly calculated by FADE [36] that is an efficient method to calculate atomic density.

*PLAST *4 and *PLAST *5 were calculated as:

$$\begin{array}{c} PLAST4=\frac{WT\text{\_}ROT4}{ATMN4\times maxASA},\\ PLAST5=\frac{WT\text{\_}ROT5}{ATMN5\times maxASA}, \end{array}$$

where *WT_ROT*4, *WT_ROT*5 count weighted rotatable single bond numbers of a residue's side chain within 4*Å *and 5*Å *respectively, and *ATMN*4, *ATMN*5 indicate the total numbers of surrounding atoms of a residue within 4*Å *and 5*Å *respectively.

### Feature selection

Feature selection is an important step in training classifiers and is often utilized to improve the performance of a classifier by removing redundant and irrelevant features.

In this work, 68 features were generated initially. Such a feature set may cause over-fitting of the model. Therefore, we employed random forests proposed by Breiman [29] to find important features, with which to get better discrimination of hot spot residues and non-hot spot residues.

Random forests are a combination of tree predictors such that each tree depends on the values of a random vector sampled independently and with the same distribution for all trees in the forests. Random forests return several measures of variable importance. The most reliable measure is based on the decrease in classification accuracy when the values of a variable in a node of a tree are permuted randomly [37].

**Figure 1:**
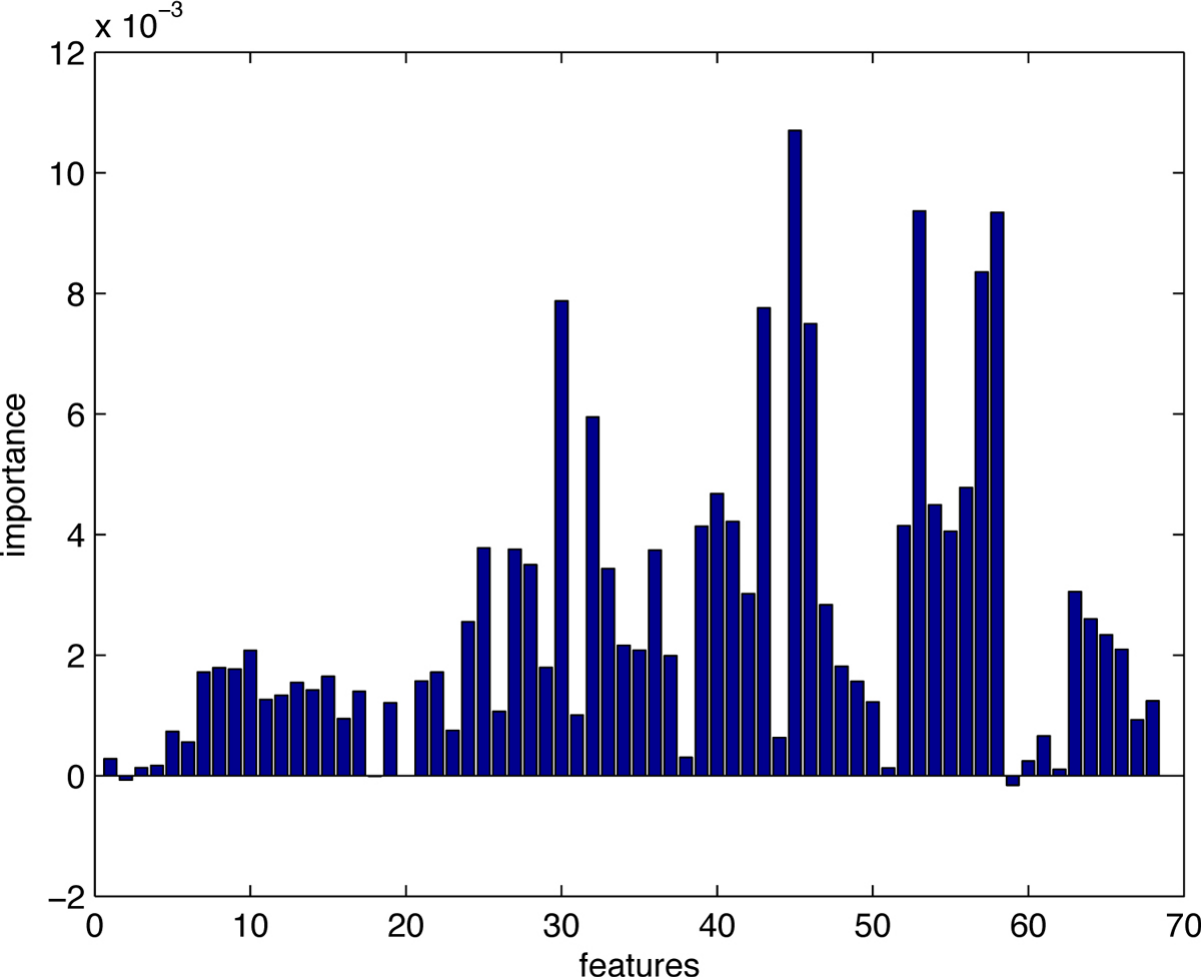
**The importance of all 68 features (*dataset*1)**. Feature importance generated by random forests. The top-10 features were picked out and various combinations were tested by 10-fold cross-validation to find the best feature subset for prediction of hot spots.

Figure 1 shows the importance of all 68 features for hot spots prediction on *dataset*1. We can clearly see how each of the features affects the accuracy of prediction. In our study, we selected the top-10 features whose values of importance are significantly higher than the others', and then tried various combinations to get the best prediction result. The features that we chose for *dataset*1 are: relative change in side-chain ASA upon complexation, relative change in side-chain mean PI upon complexation, CORE_RIM, SA_RATIO5, total RASA, DELTA_TOT.

**Figure 2:**
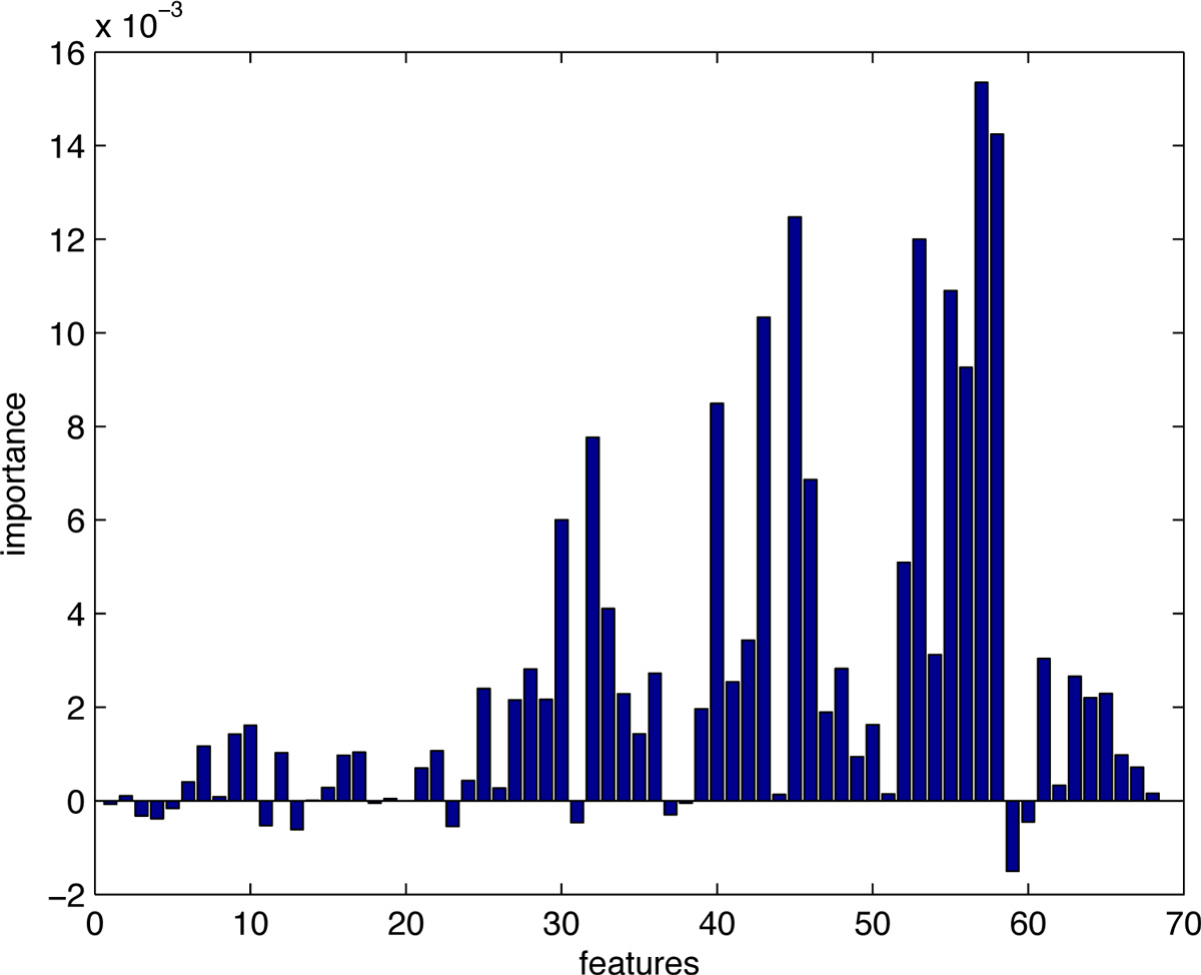
**The importance of all 68 features (*dataset*2)**. Feature importance generated by random forests. The top-10 features were picked out and various combinations were tested by 10-fold cross-validation to find the best feature subset for prediction of hot spots.

The feature importance of the balanced training data set, *dataset*2, is illustrated in Figure 2. Here, we still tried various combinations from the top-10 features. The features we used in the prediction model for *dataset*2 are: SA_RATIO5, relative change in side-chain mean PI upon complexation, relative change in minimal PI upon complexation, relative change in total ASA upon complexation, s-chain RASA, relative change in polar ASA upon complexation.

### SemiBoost framework

Mallapagada et al., [38] presented a boosting framework for semi-supervised learning to improve supervised learning, termed as *SemiBoost*, by using both labeled data and unlabeled data in the learning process. The framework is given as follows.

Given a data set *D *= {*x*_1_, *x*_2_, *x*_3_, . . ., *n_n_*}, the labels for the entire dataset can be denoted as *y *= [*y_l_*; *y_u_*] where the labeled subset is denoted by $y_{l}=\left(y_{1}^{l},\;y_{2}^{l},\;\ldots ,\;y_{n_{l}}^{l}\right)$ and the unlabeled subset is denoted by $y_{u}=\left(y_{1}^{u},\;y_{2}^{u},\;\ldots ,\;y_{n_{u}}^{u}\right)$ with *n *= *n_l _*+ *n_u_*. It can be assumed that an unlabeled data *x_u _*and a labeled data with the highest similarity to *x_u _*may share the same label. The symmetric matrix *S^lu ^*represents the similarity between labeled and unlabeled data. The term *F_l_*(**y**, *S^lu^*) stands for the inconsistency between labeled and unlabeled data. It can also be assumed that two unlabel data points with the highest similarity may share the same label. The symmetric matrix *S^uu ^*represents a similarity matrix based on the unlabeled data. The term *F_u_*(**y***_u_*, *S^uu^*) stands for the inconsistency among unlabeled data. Thus an objective function *F*(**y**, *S*) can be obtained from the above two terms. Our goal is to find the label *y_u _*that minimizes *F*(**y**, *S*).

Concretely, the objective function is given as

(1) $$F\left(y,S\right)=F_{l}\left(y,S^{lu}\right)+CF_{u}\left(y_{u},S^{uu}\right)$$

where *C *weights the importance between the labeled and unlabeled data. The two terms in (1) are given as follows:

(2) $$F_{l}\left(y,S^{lu}\right)=\overset{n_{l}}{\underset{i=1}{\sum }}\overset{n_{u}}{\underset{j=1}{\sum }}S_{i,j}^{lu}exp\left(-2y_{i}^{l}y_{j}^{u}\right),$$

(3) $$F_{u}\left(y_{u},S^{uu}\right)=\overset{n_{u}}{\underset{i,j=1}{\sum }}S_{i,j}^{uu}exp\left(y_{i}^{u}-y_{j}^{u}\right).$$

Let *h^t^*(*x*) denote the classifier trained at the *t*-th iteration by the underlying learning algorithm *A *and *H*(*x*) denote the combined classifier, we have

(4) $$H\left(x\right)=\overset{T}{\underset{t=1}{\sum }}\alpha_{t}h^{t}\left(x\right)$$

where *α_t _*is the combination weight. Then, the learning problem is transformed to the following optimization problem:

(5) $$\begin{array}{c} \text{arg}\underset{h\left(x\right),\alpha }{\text{min}}\;\;\sum_{i=1}^{n_{l}}\sum_{j=1}^{n_{u}}S_{i,j}\text{exp}\left(-2y_{i}^{l}\left(H_{j}+\alpha h_{i}\right)\right)\\ \;\;\;\;\;+C\sum_{i,j=1}^{n_{u}}S_{i,j}\text{exp}\left(H_{i}-H_{j}\right)\text{exp}\left(\alpha \left(h_{i}-h_{j}\right)\right)\\ \;\;\;\;\;\;\;\;s.t.\;h\left(x_{i}\right)=y_{i}^{l},i=1,\ldots ,n_{l}. \end{array}$$

By variable substitution and regrouping, (5) can be transformed into

(6) $$\bar{F_{1}}=\overset{n_{u}}{\underset{i=1}{\sum }}exp\left(-2\alpha h_{i}\right)p_{i}+exp\left(2\alpha h_{i}\right)q_{i}$$

where

(7) $$p_{i}=\overset{n_{l}}{\underset{j=1}{\sum }}S_{i,j}^{ul}e^{-2H_{j}}\delta \left(y_{j},1\right)+\frac{C}{2}\overset{n_{u}}{\underset{j=1}{\sum }}S_{i,j}^{uu}e^{H_{j}-H_{i}},$$

(8) $$q_{i}=\overset{n_{l}}{\underset{j=1}{\sum }}S_{i,j}^{ul}e^{-2H_{j}}\delta \left(y_{j},-1\right)+\frac{C}{2}\overset{n_{u}}{\underset{j=1}{\sum }}S_{i,j}^{uu}e^{H_{i}-H_{j}}.$$

Above, *p_i _*and *q_i _*are considered as the confidences in classifying the unlabeled data into the positive and negative classes respectively.

The SemiBoost algorithm starts with an empty ensemble. At each iteration, it computes the confidence for unlabeled data and then assigns the pseudo-labels according to both the existing ensemble and the similarity matrix. The most confident pseudo-labeled data are combined with the labeled data to train a classifier using the supervised learning algorithm. The ensemble classifier is updated by the former classifiers with appropriate weights, and the iteration is stopped when *α <*0, here

$$\alpha =\frac{1}{4}ln\frac{\sum_{i=1}^{n_{u}}p_{i}\delta \left(h_{i},1\right)+\sum_{i=1}^{n_{u}}q_{i}\delta \left(h_{i},-1\right)}{\sum_{i=1}^{n_{u}}p_{i}\delta \left(h_{i},-1\right)+\sum_{i=1}^{n_{u}}q_{i}\delta \left(h_{i},1\right)}.$$

Mallapagada et al. proved the performance improvement on the supervised algorithms by using SemiBoost on different datasets, and SemiBoost outperforms the benchmark semi-supervised algorithms [38].

### SVM

In this paper, we employed the support vector machine (SVM) as the underlying supervised learning algorithm in the SemiBoost framework.

SVM was first developed by Vapnik [39] and was originally employed to find a linear separating hyperplane that maximizes the distance between two classes. SVM can deal with the problems that can not be linearly separated in the original input space by adding a penalty function of violation of the constraints to the optimization criterion or by transforming the input space into a higher dimension space. It was widely used for developing methods in Bioinformatics and has been proved to be effective in predicting hot spots [27,28,30].

### sbSVM: an SVM with semi-supervised boosting to predict hot spots

In this study, we propose a new method that combines the semi-supervised boosting framework with the underlying supervised learning algorithm SVM to predict hot spots.

In the original SemiBoost framework proposed by Mallapagada et al., both confidence values of *p_i _*and *q_i _*might be large and there no any persuasive criterion to choose the most confident unlabeled data. Directly choosing the top 10% of the unlabeled data will include too many ambiguous samples with pseudolabel at the early iterations.

In order to overcome the above problem, we modified the terms in Equation (2) and Equation (3) by assigning weights according to the similarity matrix *S^ul ^*and *S^uu ^*as follows:

(10) $$\begin{array}{c} \text{arg}\underset{h\left(x\right),\alpha }{\text{min}}\phi \overset{n_{u}}{\underset{i=1}{\sum }}\frac{\sum_{j=1}^{n_{l}}S_{i,j}^{ul}\text{exp}\left(-2y_{i}^{l}\left(H_{i}+\alpha h_{i}\right)\right)}{\underset{j}{\sum }S_{i,j}^{ul}}\\ \;\;\;+\psi \overset{n_{u}}{\underset{i=1}{\sum }}\frac{\sum_{j=1}^{n_{u}}S_{i,j}^{uu}\text{exp}\left(H_{j}-H_{i}\right)\text{exp}\left(\alpha \left(h_{j}-h_{i}\right)\right)}{\underset{j}{\sum }S_{i,j}^{uu}}\\ s.t.\;h\left(x_{j}\right)=y_{j}^{l},j=1,\ldots ,n_{l} \end{array}$$

where $\phi =1/\left(1+\frac{C}{2}\right)$ and $\psi =C/\left(1+\frac{C}{2}\right)$. *C *is the tuning parameter for the importance of the labeled and unlabeled data, and we set its default value to *n_l_/n_u_*. Given the above function, we can obtain the values of *p_i _*and *q_i _*as follows:

(11) $$p_{i}=\frac{1}{1+\frac{C}{2}}\overset{n_{l}}{\underset{j=1}{\sum }}S_{i,j}^{ul}e^{-2H_{j}}\delta \left(y_{j},1\right)+\frac{\frac{C}{2}}{1+\frac{C}{2}}\overset{n_{u}}{\underset{j=1}{\sum }}S_{i,j}^{uu}e^{H_{j}-H_{i}},$$

(12) $$q_{i}=\frac{1}{1+\frac{C}{2}}\overset{n_{l}}{\underset{j=1}{\sum }}S_{i,j}^{ul}e^{-2H_{j}}\delta \left(y_{j},-1\right)+\frac{\frac{C}{2}}{1+\frac{C}{2}}\overset{n_{u}}{\underset{j=1}{\sum }}S_{ij}^{uu}e^{H_{i}-H_{j}},$$

which will have the maximum of 1. Then we sample the unlabeled data according to the following two criteria: (1) |*p_i _*− *q_i_*| ≥ 0.3, (2) Top 10% |*p_i _*− *q_i_*|. With that, we can assign pseudolabels to unlabeled data according to *sign*(*p_i _*− *q_i_*), and choose the most credible ones for training the classifier.

**Figure 3:**
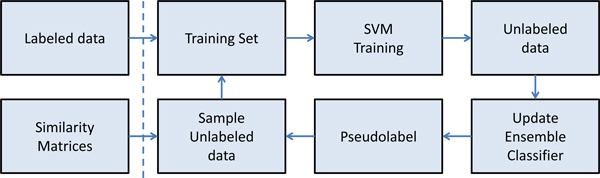
**The workflow of sbSVM**. The labeled data is input and similarity matrices are calculated before the iteration. During each iteration, some of the unlabeled data that have the highest classification confidence will be sampled into the training dataset for the next iteration.

At each iteration, like the original SemiBoost framework, we update the ensemble classifier *H*(*x*) with *H*(*x*) + *α_t_h_t_*(*x*). The algorithm stops when the number of iterations reaches *T *(a predefined parameter) or *α <*0. Figure 3 illustrates the basic workflow of the sbSVM approach. The similarity matrices are calculated initially and play an important role in selecting unlabeled samples. The unlabeled data with highest confidence will be added to the training set for the next iteration of training.

### Performance evaluation

To evaluate the classification performance of the method sbSVM proposed in this study, we adopted some widely used measures, including *precision*, *recall *(*sensitivity*), *specificity*, *accuracy *and *F1 *score. These measures are defined as follows:

$$\begin{array}{c} Precision=\frac{TP}{\left(TP+FP\right)},\\ Recall\left(sensitivity\right)=\frac{TP}{\left(TN+FP\right)},\\ Specificity=\frac{TN}{\left(TN+FP\right)},\\ Accuracy=\frac{\left(TP+TN\right)}{\left(TP+FP+TN+FN\right)},\\ F1=2\times \frac{Precision\times Recall}{Precision+Recall}. \end{array}$$

Here, *TP*, *FP*, *TN *and *FN *denote the numbers of true positives (correctly predicted hot spot residues), false positives (non-hot spot residues incorrectly predicted as hot spots), true negatives (correctly predicted non-hot spot residues) and false negatives (hot spot residues incorrectly predicted as non-hot spot residues), respectively. *F1 *score is a composite measure, which is widely used to evaluate prediction accuracy considering both *precision *and *recall*.

## Results and discussion

### Parameter selection

The similarity matrices *S^ul ^*and *S^uu ^*are computed by the radial basis function. For example, let *x_i _*and *x_j _*be two samples from the dateset, the similarity between them is calculated by *S_i,j _*= exp(− (**x***_i _*− **x***_j_*)^2^*/*2*σ*^2^), where *σ *is the scale parameter that has a great impact on the performance of the learning algorithm. We tested 10 values of *σ *from 1 to 10 in a 10-fold cross-validation on *dataset*1 to get the best performance of our method. The performance of our method varies according to the value of *σ*, which is listed in Table 3. We chose the value of 3 for *σ *that produces the best performance. And for *dataset*2, our method has the best performance when *σ *is set to 1.

**Table 3:** The performance of sbSVM when *σ *changes from 1 to 10 with stepsize = 1 (cross-validation on *dataset*1).

Recall	Precision	Specificity	Accuracy	F1	*σ*
0.82	0.48	0.72	0.74	0.61	1-2

0.82	0.5	0.74	0.76	0.62	3

0.82	0.47	0.7	0.73	0.6	4-6

0.82	0.47	0.7	0.75	0.6	7-9

0.82	0.48	0.71	0.74	0.6	10

The optimization process will stop when *α <*0 during the iterations. However, in order to avoid a slow convergence, we set the maximum number of iterations *T *= 20.

### Performance comparison and cross-validation

**Figure 4:**
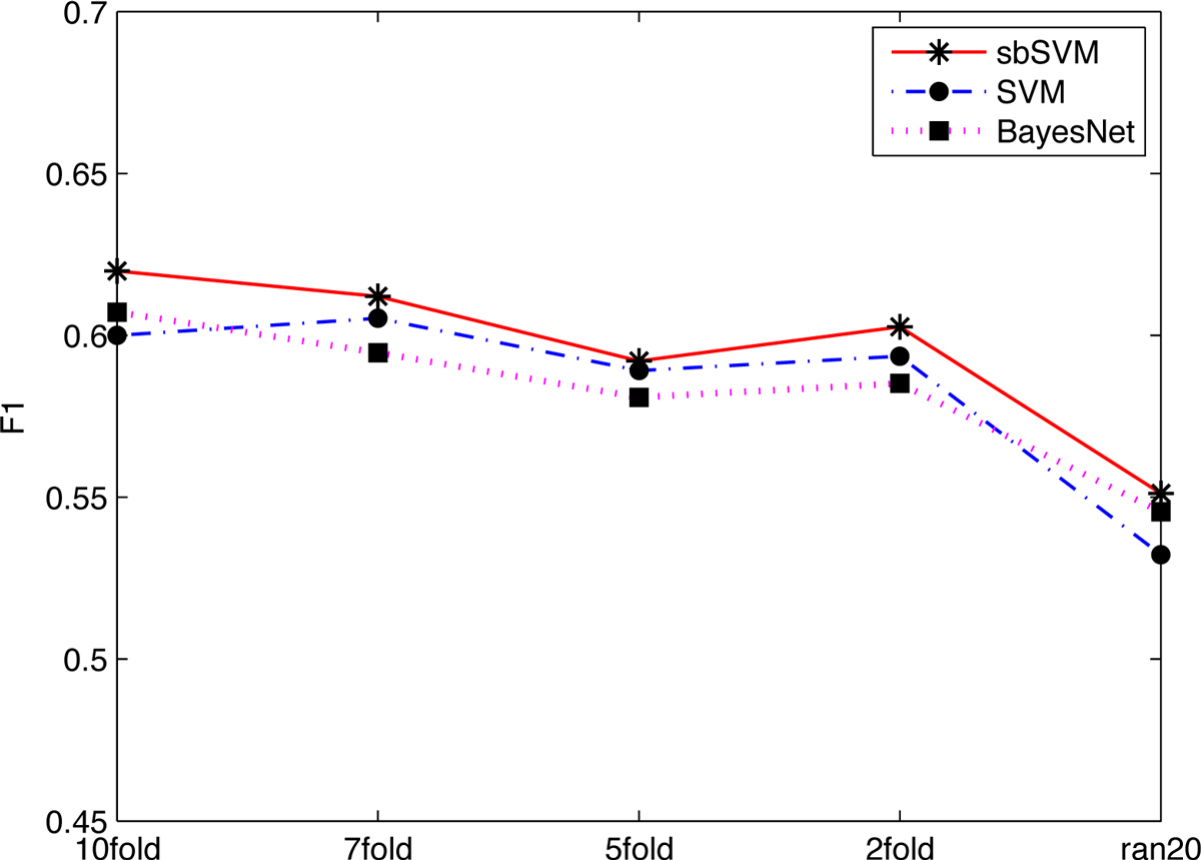
**The comparison of different methods by cross-validation**. Among all methods, sbSVM has the highest *F1-score*. sbSVM improves the prediction performance even when the training dataset is small.

In this section, the performance of sbSVM is examined and compared with three existing machine learning methods, including SVM [39], Bayes network [40] and decision tree C4.5 [41]. We first conducted several cross-validation (10/7/5/2-folds) tests and an additional test called *random*-20 test (where we randomly chose 20 samples from the training dataset to train the predictor and then perform prediction on the remaining data. This process was repeated 10 times to get the averaged result) on *dataset*1 to show that the boosting with unlabeled data method, sbSVM, outperforms the other three methods. The experimental results (F1 scores) are shown in Figure 4. From Figure 4, we can see that even when the training data is small, sbSVM still outperforms the others. As all the results of decision tree are less than 0.45, we do not show them in Figure 4.

Our approach was further compared with other five existing hot-spot prediction methods by 10-fold cross-validation on *dataset*1. The compared methods include KFC [25], Robetta [21], FOLDEF [22], MIN-ERVA [26] and KFC2 [28].

**Figure 5:**
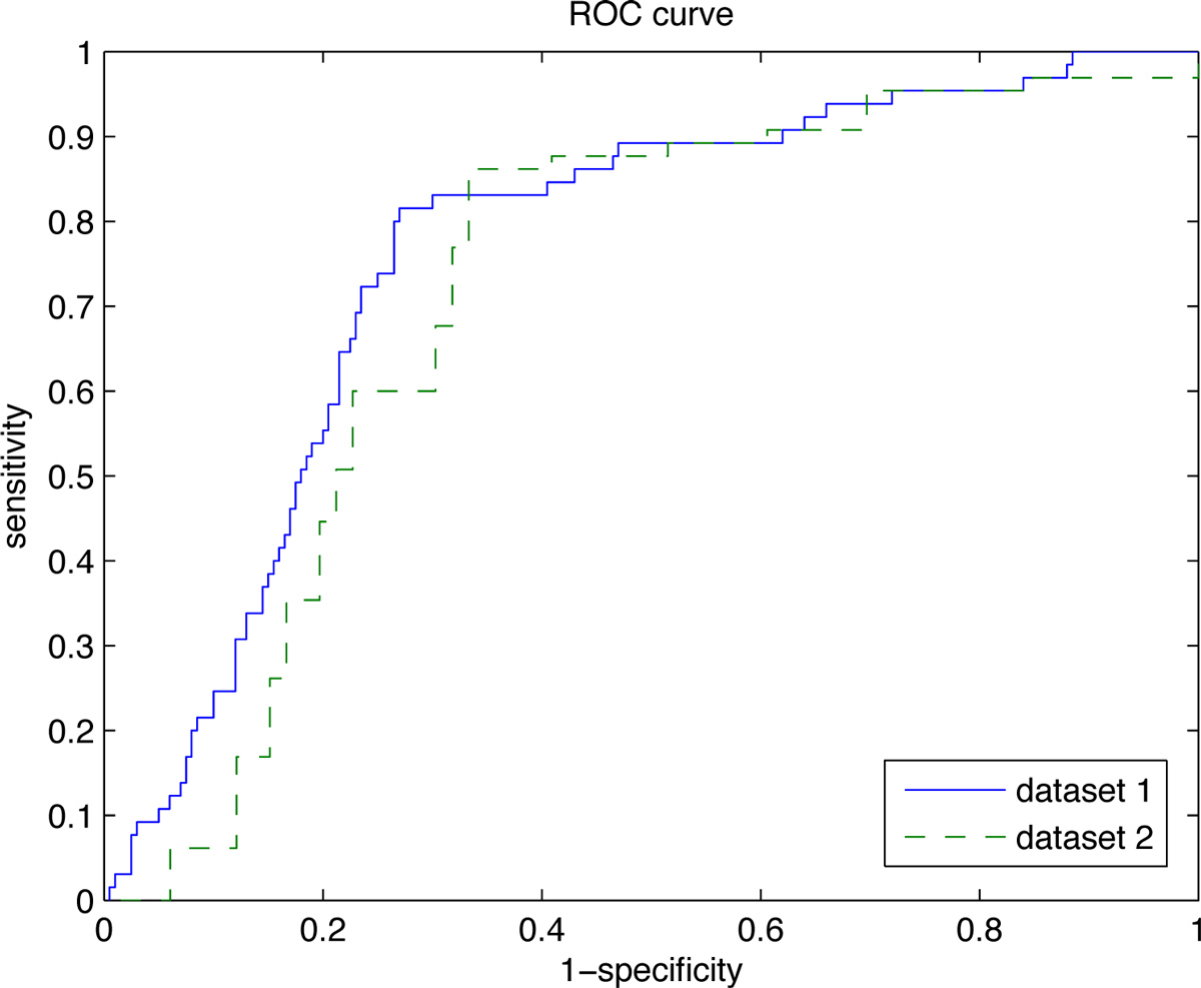
**ROC curves of sbSVM on *dataset *1 and *dataset *2**. The area under the curves are 0.764 (*datset *1) and 0.758 (*dataset *2).

The results of the methods compared were collected from the original papers where these methods were published. All results are listed in Table 4. We can see that sbSVM has the best *recall *of 0.82 among all these methods, and its *F1-score *is only outperformed by MINERVA. Besides, the *specificity *and *accuracy *of our method are also competitive. Table 5 shows the results of 10-fold cross-validation on *dataset*2. We can see that our method has outstanding performance, with the highest *recall *(0.89) and *F1 *score (0.80). Figure 5 illustrates the ROC curves of our method on both datasets. The area under the curves are 0.764 (*datset*1) and 0.719 (*dataset*2).

**Table 4:** The cross-validation results on *dataset*1.

Methods	Recall	Precision	Specificity	Accuracy	F1
KFC	0.55	0.58	0.85	0.78	0.56

Robetta	0.49	0.62	0.9	0.8	0.55

FOLDEF	0.32	0.59	0.93	0.78	0.41

MINERVA	0.58	0.73	0.89	0.82	0.65

sbSVM	0.82	0.5	0.74	0.76	0.62

**Table 5:** The cross-validation results on *dataset*2.

Methods	Recall	Precision	Specificity	Accuracy	F1
KFC	0.55	0.81	0.88	0.70	0.66

Robetta	0.51	0.8	0.88	0.7	0.62

FOLDEF	0.31	0.8	0.93	0.62	0.44

MINERVA	0.58	0.93	0.96	0.77	0.72

KFC2	0.78	0.77	0.78	0.78	0.78

sbSVM	0.89	0.73	0.68	0.79	0.8

### Independent test

Here we evaluate sbSVM and compare it with other methods by independent test on *ind*-*dataset *described in the *Method *section. The results are presented in Table 6 and Table 7. Performance results of the compared methods were obtained from their corresponding web servers.

**Table 6:** Independent test results (sbSVM was trained on *dataset*1).

Methods	Recall	Precision	Specificity	Accuracy	F1
KFC	0.31	0.48	0.85	0.69	0.38

Robetta	0.33	0.52	0.87	0.71	0.4

FOLDEF	0.26	0.48	0.88	0.69	0.34

MINERVA	0.44	0.65	0.9	0.76	0.52

sbSVM	0.77	0.46	0.6	0.66	0.58

**Table 7:** Independent test results (sbSVM was trained on *dataset*2).

Methods	Recall	Precision	Specificity	Accuracy	F1
KFC	0.33	0.42	0.79	0.65	0.37

Robetta	0.39	0.58	0.87	0.72	0.46

FOLDEF	0.26	0.48	0.87	0.69	0.33

MINERVA	0.46	0.69	0.91	0.77	0.55

KFC2	0.74	0.56	0.74	0.74	0.64

sbSVM	0.82	0.51	0.64	0.70	0.63

Table 6 shows that when our method sbSVM was trained on *dataset*1 and tested on *ind*-*dataset*, we obtain the highest *recall *(0.77) and *F1 *score (0.58).

Table 7 demonstrates that when our method was trained on the balanced dataset *dataset*2 and tested on *ind*-*dataset*, our method still get the highest *F1 *score (0.64), and its other measures, *recall *(0.72), *specificity *(0.77) and *accuracy *(0.76) are still competitive among all tested methods.

### Remarks on the selected features

In this paper, we extracted a large set of features from previous studies, but only several were used in hot-spot prediction. The selected features for *dataset*1 and *dataset*2 are listed in Table 8. Note that none of the sequence features were chosen in the two final feature combinations for *dataset*1 and *dataset*2. This may imply that general sequence information is not so important in hot spot prediction.

**Table 8:** Selected features for dataset1 and dataset2.

Selected features for *dataset*1	Selected features for *dataset*2
relative change in side-chain ASA upon complexation	SA_RATIO5

relative change in side-chain mean PI upon complexation	relative change in side-chain mean PI upon complexation

*CORE_RIM*	relative change in minimal PI upon complexation

*SA_RATIO*5	relative change in total ASA upon complexation

total RASA	s-chain RASA

*DELTA_TOT*	relative change in polar ASA upon complexation

The relative change in side-chain ASA upon complexation, the relative change in total ASA upon complexation, SA_RATIO5 and CORE_RIM measure from different aspects the changes in accessible surface of a residue between unbound and bound states. These structural features were all chosen in our prediction, which suggests that residues surrounded by others and sheltered from solvents are more likely to be hot spots [17]. Meanwhile, the two different relative changes in Protrusion Index (relative change in side-chain mean PI upon complexation and relative change in minimal PI upon complexation) used in our method are also strong evidence of hot spots. It was found that hot spots tend to protrude into complementary pockets [17]. Therefore, these selected structural features also suggest that the high local packing density of a residue is helpful in predicting hot spots [42].

As the structural information used in this paper indicate the nature of hot spots, our approach obtained the highest recall in hot spot prediction.

### Case study

EPO (Erythropoietin) is produced by interstitial fibroblasts in the kidney, which is in close association with peritubular capillary and tubular epithelial cells. It is the hormone that regulates red blood cell production.

There exists a competition between EMP1 (pdbID:1ebp, chainC) and EPO to bind the erythropoietic receptor (EPOR) (pdbID:1ebp, chainA) [43]. Experimentally found hot spots at the 1ebpAC interface are F93A, M150A, F205A and W13C, and T151A, L11C and T12C were found experimentally to be non-hot spots (in BID). Our method predicts correctly two out of the four hot spots - M150A and F205A, and all of the three non-hot spots.

**Figure 6:**
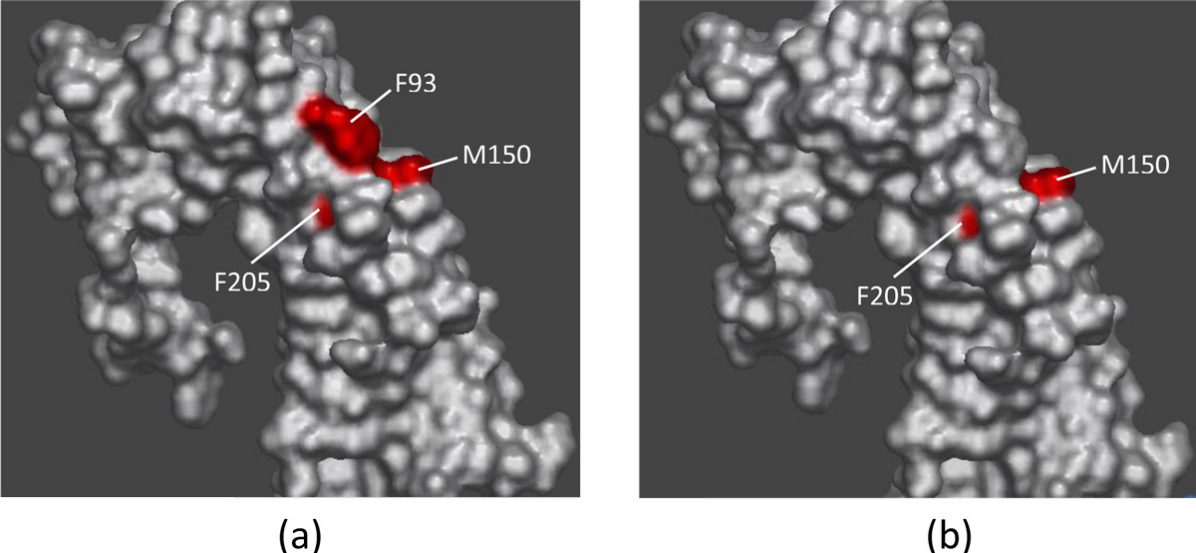
**A case study**. The visualization of prediction results on chain A of EMP1. Red color indicates hot spots. (a) Physical experimental results; (b) Computational results predicted by our method sbSVM.

Figure 6(a) shows the experimental results on chain A of EMP1. Red color indicates the residues F93A, M150A and F205A, which were found to be hot spots. Figure 6(b) shows the prediction results of our method sbSVM on chain A. Here, red color shows the hot spots M150A and F205A.

## Conclusions

In this study we proposed a new effective computational method, named sbSVM, to identify hot spots at the protein interfaces. We combined sequence and structure features, and selected the most important features by random forests. Our method is based on a semi-supervised boosting framework that samples some useful unlabeled data at each iteration to improve the performance of the underlying classifier (SVM in this paper). The performance of sbSVM was evaluated by 10-fold cross-validation and independent test. Results show that our approach, with the best sensitivity and F1 score, can provide better or at least comparable performance than or to the major existing methods, including KFC, Robetta, FOLDEF, MINERVA and KFC2.

Our study has achieved substantial improvement on performance of hot spots prediction by using the unlabeled data. In our future work, on the one hand we will explore more useful features of both hot spots and non-hot spots, and on the other hand, we will try to develop more sophisticated hot spot prediction methods based on advanced machine learning techniques (e.g., transfer learning and spare representation).

## Authors' contributions

BX and LD designed the method, BX implemented the method, conducted the experiments and data analysis, and finished the draft. XW prepared the data. SZ and JG conceived the work, supervised the research and revised the manuscript.

## Competing interests

The authors declare that they have no competing interests.
